# Inside-Out Mechanism of Referred Visceral Pain: Protocol for a Systematic Review of Cutaneous Neurogenic Inflammation in Preclinical Animal Models of Visceral Diseases

**DOI:** 10.2196/67852

**Published:** 2026-04-17

**Authors:** EunMee Yang, Dennis Muñoz-Vergara, Andrew C Ahn, Weidong Lu, Vitaly Napadow, Alexandria E Cronin, Peter M Wayne

**Affiliations:** 1 Osher Center for Integrative Health, Brigham and Women’s Hospital and Harvard Medical School Boston, MA United States; 2 Department of Medical Oncology, Dana-Farber Cancer Institute and Harvard Medical School Boston, MA United States; 3 Department of Physical Medicine and Rehabilitation, Spaulding Rehabilitation Hospital and Harvard Medical School Boston, MA United States; 4 Department of Radiology, Athinoula A Martinos Center for Biomedical Imaging and Harvard Medical School Charlestown, MA United States; 5 Countway Library of Medicine, Harvard Medical School Boston, MA United States

**Keywords:** referred visceral pain, cutaneous neurogenic inflammation, Evans Blue dye, somatic hypersensitivity, systematic review protocol, preclinical studies, neurogenic, inflammation, visceral pain, pain, hypersensitivity, animal models, systematic review, review, visceral diseases, somatic pain, sensitivity, sensitive, animal

## Abstract

**Background:**

Visceral diseases often lead to referred pain, but clinical management is challenging due to poor understanding of the underlying mechanisms. Prior preclinical studies have shown that experimental induction of visceral diseases in animal models can cause cutaneous neurogenic inflammation, as assessed by Evans Blue dye extravasation. Neurogenic inflammation is associated with increased pain sensitivity. Thus, these preclinical findings may provide important mechanistic insights into the relationship between visceral diseases and referred somatic pain.

**Objective:**

The objective of this systematic review is to synthesize evidence on cutaneous neurogenic inflammation and corresponding somatic pain sensitivity in preclinical animal models of visceral diseases, to lay out the current state of knowledge, and to identify any gaps or limitations to be addressed in future research on this topic.

**Methods:**

This systematic review will be conducted, following the PRISMA-P (Preferred Reporting Items for Systematic Review and Meta-Analysis Protocol) guidelines. The literature search will be conducted in MEDLINE (Ovid), Embase, and Web of Science, using search terms related to cutaneous neurogenic inflammation and animal models of visceral diseases. Two investigators will independently screen and select studies meeting the eligibility criteria. For each included study, a predefined template will be used to extract relevant information, including the details of animal species and strains, visceral disease models, outcomes related to the distribution and extent of Evans Blue dye extravasation, and other pertinent findings and methodologies. Quality assessments will be independently performed by two investigators using the Risk of Bias tool from the Systematic Review Centre for Laboratory Animal Experimentation (SYRCLE). A qualitative summary of the findings will be provided for outcomes related to the extent and distribution of Evans Blue dye extravasation.

**Results:**

As of August 2025, a total of 536 records were identified from database search, and 13 studies met the eligibility criteria to be included in the review. Results from data extraction and quality assessments are expected to be published in fall 2026.

**Conclusions:**

Prior preclinical animal studies have investigated cutaneous neurogenic inflammation as a potential mechanistic outcome of referred visceral pain. The proposed review will be the first to summarize research findings and study quality on this topic. This will be an important step toward gaining insights into one of the potential mechanisms underlying referred visceral pain and informing future research in this area.

**International Registered Report Identifier (IRRID):**

DERR1-10.2196/67852

## Introduction

Referred visceral pain is a common yet poorly understood phenomenon, whereby pain originating from internal organs is perceived as pain in other areas of the body, often in somatic structures such as the skin [1-3]. Examples include pain in the chest, left arm, neck, jaw, and upper back during myocardial infarction, as well as abdominal or back pain in pancreatitis, gastrointestinal disorders, and interstitial cystitis [4-12]. In clinical practice, management of referred visceral pain remains challenging because currently available treatments offer limited relief, and its underlying mechanisms are not fully understood [5,13].

To date, explanations for referred visceral pain have focused on central mechanisms, particularly the concept of viscerosomatic convergence. In this model, visceral and somatic afferents converge onto shared spinal neurons, causing the central nervous system to misinterpret visceral pain as originating from somatic regions [13]. While this framework explains the central integration of visceral and somatic signals, it does not account for any physiological changes observed in the somatic structures.

Emerging evidence from preclinical animal studies suggests an additional mechanism involving cutaneous neurogenic inflammation [14-20]. This process involves crosstalk between visceral and somatic afferents, whereby noxious input from the viscera activates the convergent somatic afferents, triggering antidromic signaling and peripheral release of vasoactive neuropeptides such as substance P and calcitonin gene-related peptide. These neuropeptides, in turn, cause localized inflammatory changes, including vasodilation and plasma extravasation and are associated with increased pain sensitivity [21,22]. Therefore, such cutaneous manifestations may represent an “inside-out” extension of referred visceral pain that contribute to somatic hyperalgesia.

In their 1997 study, Wesselmann and Lai [14] provided the first key experimental evidence for this mechanism by demonstrating that uterine inflammation in rats induced neurogenic inflammation on the body surface along the referred area of somatotopic overlap. Upon intravenous injection of Evans Blue (EB) dye, plasma extravasation was visualized as blue-stained spots on the skin. Subsequent studies have applied this approach (ie, intravenous injection of EB dye) to models of other visceral diseases, reporting segmentally organized patterns of EB dye extravasation [15-20]. Despite these important findings, to date, no systematic or narrative review has been conducted to summarize the preclinical evidence investigating cutaneous neurogenic inflammation in visceral disease models.

Understanding the relationship between cutaneous neurogenic inflammation and visceral diseases has important translational implications for pain research. Unlike central models that primarily explain altered pain perception, evidence of peripheral physiological changes—such as increased microvascular permeability and inflammation in the skin—suggests a measurable biological correlate of referred visceral pain. Characterizing these changes may provide objective biomarkers of viscerosomatic interactions and improve understanding of how visceral disease may give rise to referred pain. Such insights could inform the development of targeted diagnostic and therapeutic strategies for managing visceral pain and its somatic manifestations.

Therefore, the purpose of this systematic review is to synthesize preclinical evidence of cutaneous neurogenic inflammation in animal models of visceral disease. Specifically, we aim to examine whether experimentally induced models of visceral disease manifest signs of cutaneous neurogenic inflammation, as assessed by EB dye extravasation, and whether there are corresponding changes in pain sensitivity. Secondary aims include assessing the methodological quality and risk of bias of the included studies, summarizing complementary laboratory findings, and identifying gaps in the existing literature to inform future research.

## Methods

### Study Design and Registration

We will conduct a systematic review of preclinical animal studies, according to the guidelines recommended in PRISMA-P (Preferred Reporting Items for Systematic Review and Meta-Analysis Protocols) [23]. A completed PRISMA-P checklist can be found as Multimedia Appendix 1. In preparing this protocol, we have also adopted an animal systematic review protocol format developed by de Vries et al [24], which was designed specifically for review of animal studies. Any changes or amendments made to the protocol will be recorded in PROSPERO as minor amendments or major revisions, following their procedures, with each update including the date, version number, and a summary of the changes. Any deviations from the registered protocol will also be justified and documented in the final manuscript.

### Search Strategy

A literature search will be conducted in MEDLINE (Ovid), Embase, and Web of Science by using terms related to the research question, including but limited to “cutaneous neurogenic inflammation” and “Evans Blue dye extravasation.” The initial search strategy was jointly developed by two investigators (EY and PW) and a medical librarian/information scientist (AC). It was then reviewed and refined based on suggestions and feedback from other co-investigators. The search will be limited to English-language publications. No limitation will be placed on the publication date range. In addition to electronic database search, we will hand search references of the included studies to identify additional relevant studies. The full search strategies for all databases can be found in Table 1. The search will be rerun prior to data synthesis to capture newly published studies, and updated search dates and strategies will be documented in the supplementary materials and reported in the final manuscript to ensure transparency and reproducibility.

**Table 1:** Database search strategy.

Database	Search strategy
MEDLINE	1. exp “Extravasation of Diagnostic and Therapeutic Materials”/ OR exp “Evans Blue”/ OR exp “Acupuncture Points”/ 2. ((Acupuncture OR neurogenic) adj2 (spot* OR point*)).ab,kf,kw,ti. 3. (acupoint* OR Neuro-Sp* OR “Evans blue” OR “EB dye” OR “dye extravasation” OR “plasma extravasation”).ab,kf,kw,ti. 4. 1 OR 2 OR 3 5. exp “Neurogenic Inflammation”/ 6. (“neurogenic inflammation” OR (visceral AND (disease OR pain OR inflam*) AND neuro*) OR (referred AND pain AND neuro*)).ab,kf,kw,ti. 7. 5 OR 6 8. 4 AND 7
Embase	1. ('contrast medium extravasation'/exp OR 'evans blue'/exp OR 'acupuncture point'/exp) AND [embase]/lim 2. (((acupuncture OR neurogenic) NEAR/2 (spot* OR point*)):ab,kw,ti) AND [embase]/lim 3. ((acupoint* OR Neuro-Sp* OR 'Evans blue' OR 'EB dye' OR 'dye extravasation' OR 'plasma extravasation'):ab,kw,ti) AND [embase]/lim 4. 1 OR 2 OR 3 5. ('neurogenic inflammation'/exp) AND [embase]/lim 6. (('neurogenic inflammation' OR (visceral AND (disease OR pain OR inflam*) AND neuro*) OR (referred AND pain AND neuro*)):ab,kw,ti) AND [embase]/lim 7. 5 OR 6 8. 4 AND 7
Web of Science	1. TI=((acupuncture OR neurogenic) NEAR/2 (spot* OR point*)) 2. AB=((acupuncture OR neurogenic) NEAR/2 (spot* OR point*)) 3. AK=((acupuncture OR neurogenic) NEAR/2 (spot* OR point*)) 4. TI=(acupoint* OR Neuro-Sp* OR “Evans blue” OR “EB dye” OR “dye extravasation” OR “plasma extravasation”) 5. AB=(acupoint* OR Neuro-Sp* OR “Evans blue” OR “EB dye” OR “dye extravasation” OR “plasma extravasation”) 6. AK=(acupoint* OR Neuro-Sp* OR “Evans blue” OR “EB dye” OR “dye extravasation” OR “plasma extravasation”) 7. #1 OR #2 OR #3 OR #4 OR #5 OR #6 8. TI=(“neurogenic inflammation” OR (visceral AND (disease OR pain OR inflam*) AND neuro*) OR (referred AND pain AND neuro*)) 9. AB=(“neurogenic inflammation” OR (visceral AND (disease OR pain OR inflam*) AND neuro*) OR (referred AND pain AND neuro*)) 10. AK=(“neurogenic inflammation” OR (visceral AND (disease OR pain OR inflam*) AND neuro*) OR (referred AND pain AND neuro*)) 11. #8 OR #9 OR #10 12. #7 AND #11

Of note, we found that some of the preclinical studies on this topic were conducted by acupuncture researchers aiming to compare the locations and distributions of cutaneous neurogenic inflammatory spots with that of acupuncture points, the specific points on the body surface that are stimulated during acupuncture treatment [15-20]. Thus, we also included terms related to “acupuncture points” to be more comprehensive in our search strategy (Table 1).

### Eligibility

A summary of the inclusion and exclusion criteria is provided in Textbox 1. Briefly, this review will include preclinical animal studies, investigating experimentally induced models of acute or chronic visceral diseases. Studies involving humans, in vitro, or ex vivo experiments will be excluded. Studies using EB dye or other validated markers of plasma extravasation will be considered for inclusion. EB is an azo dye that binds strongly to blood albumin, and its leakage into the surrounding tissue can be visualized to assess the extent and distribution of cutaneous neurogenic inflammation [25,26]. Only studies with a control or baseline comparison and sufficient methodological detail for data extraction will be included.

**Inclusion and exclusion criteria.**

**Inclusion criteria**

- Preclinical animal studies using acute or chronic visceral disease models
- Inclusion of a comparison condition
- Use of Evans Blue dye or equivalent validated measures of plasma extravasation to assess cutaneous neurogenic inflammation
- Studies reporting original data in peer-reviewed journals
- Published in English with sufficient methodological detail for data extraction

**Exclusion criteria**

- Human studies, clinical trials, or in vitro/ex vivo experiments
- Studies lacking a control or baseline condition for comparison with the disease model
- Studies not using Evans Blue dye or other equivalent validated measures of plasma extravasation
- Reviews, commentaries, editorials, conference abstracts, theses, preprints, or duplicated datasets
- Non-English publications or reports with incomplete methodological information preventing reliable data extraction

### Study Selection

Two investigators (EY and DMV) will independently screen all the studies resulting from the literature search to include relevant studies that meet the predefined inclusion and exclusion criteria. Screening will first be conducted based on titles and abstracts. Studies that do not meet the eligibility criteria will be excluded at this stage. The remaining studies will be screened by retrieving and examining the full texts for eligibility. Interrater agreement will be assessed using Cohen κ. Any disagreements between reviewers will first be discussed to reach a consensus; if unresolved, they will be adjudicated by a third investigator (PW).

Duplicate publications and overlapping datasets will be identified by cross-checking study details (eg, author names, animal models, experimental methods). When multiple reports describe the same dataset, the most comprehensive or recent publication will be included, and additional information from other reports will be extracted as appropriate to avoid duplication.

### Data Extraction

Two investigators (EY and DMV) will independently extract data from all included studies, using a predefined template (Multimedia Appendix 2). Data extraction will first be piloted on a subset of studies to ensure consistency and completeness between reviewers. Any discrepancies during data extraction will be resolved through discussion and consensus; if unresolved, they will be adjudicated by a third investigator (PW).

Briefly, the data extraction table includes the following: (1) publication details (eg, title, authors, publication year); (2) study population (eg, details on animal species and strains, sample size, and other characteristics of the animals such as sex and age); (3) visceral disease model, including the details of the methodology used to induce the disease model (eg, name and dosage of chemical irritant used to induce the disease model); (4) comparison or control group (eg, use of saline); (5) outcomes measures (eg, comparisons of EB dye extravasation in disease and control groups in terms of distribution and numbers of blue dots), including the details of the methodology (eg, dosage and route of EB dye administration); (6) outcomes and methods relating to pain sensitivity assessment of blue dots; (7) any other relevant methods and results from included studies, which provide additional insights about the neurocircuitry of referred visceral pain as well as other pathophysiological features of the skin in visceral diseases; and (8) if applicable, pertinent information about acupuncture point(s) (eg, names of acupuncture points in close proximity to blue dots).

The primary outcome of this review will be the extent of cutaneous neurogenic inflammation in response to experimental induction of visceral disease, as assessed by the distribution and extent of EB dye extravasation. Secondary outcomes will include pain sensitivity assessments as well as additional measures of neurogenic inflammation such as immunohistochemistry for neuropeptides (eg, substance P, calcitonin gene-related peptide), if available. Acupuncture point-related data will also be considered secondary and extracted only when directly relevant to the main mechanistic question.

### Risk of Bias Assessment

The included studies will be independently assessed for their methodological quality and risk of bias by two investigators (EY and DM-V). Given that this review involves preclinical animal studies, we will adopt the Risk of Bias (RoB) tool from the Systematic Review Centre for Laboratory Animal Experimentation (SYRCLE), which has been developed to address the specific challenges and complexities associated with assessing the rigor of evidence derived from animal research [24,27]. Briefly, SYRCLE’s RoB tool includes 10 entries related to 6 types of bias: selection bias, performance bias, detection bias, attrition bias, reporting bias, and other biases. This tool is designed to be applicable across different animal species and experimental models. Using this RoB tool, we will identify any potential limitations in the design, conduct, and reporting of included studies and determine the overall strength of evidence regarding cutaneous neurogenic inflammation in visceral diseases. Both reviewers have previously been trained in the use of the SYRCLE risk of bias tool and will independently assess the included studies and then discuss their evaluations to reach consensus. Any disagreements will be resolved through discussion and consensus or review; if unresolved, they will be adjudicated by a third investigator (PW).

### Data Synthesis

The findings of individual studies included in this review will be synthesized to identify overall patterns and draw conclusions relevant to the research question, taking into account the methodological quality of the included studies. For outcomes related to the distribution and extent of EB dye extravasation, a descriptive synthesis will be performed to summarize key trends across models, including species, visceral organ involved, and induction method. Given the anticipated heterogeneity in study design, disease induction, and outcome quantification, a meta-analysis will not be planned at this stage. Instead, findings will be reported narratively, highlighting consistent observations and methodological differences among studies. If sufficient homogeneity and quantitative data become available, a meta-analysis may be considered in future analyses.

## Results

**Figure 1:**
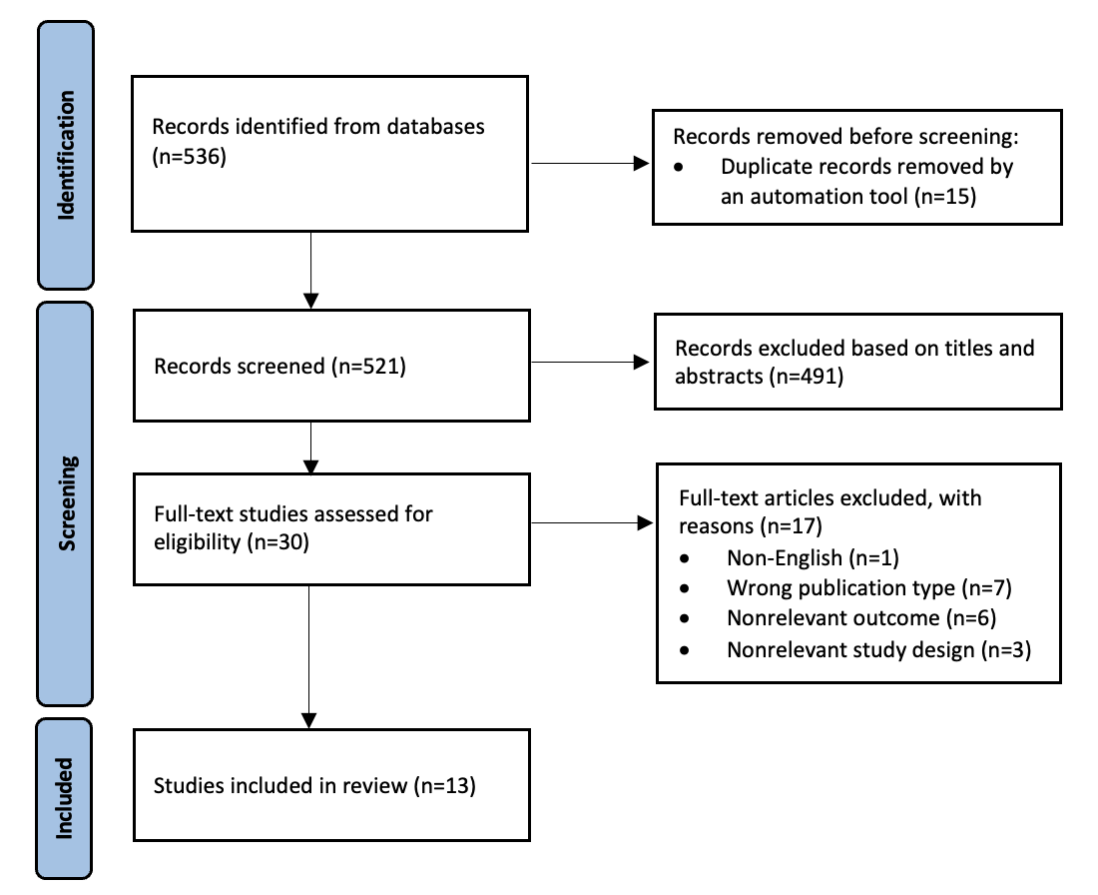
PRISMA (Preferred Reporting Items for Systematic Reviews and Meta-Analyses) flow diagram.

As of August 2025, a total of 536 records were identified from database search, and 13 studies met the eligibility criteria to be included in the review (Figure 1). Results from data extraction and quality assessment are expected to be published in fall 2026.

## Discussion

To date, several preclinical studies have investigated cutaneous neurogenic inflammation as a potential mechanistic correlate of referred visceral pain, yet no synthesis of the collective evidence has been performed. To our knowledge, this proposed review will be the first to systematically search, summarize, and evaluate the quality of relevant studies on this topic.

This protocol was designed to ensure methodological rigor, transparency, and reproducibility. It follows the PRISMA-P guidelines, with predefined eligibility criteria, data extraction procedures, and a risk-of-bias assessment using the SYRCLE tool. These measures are intended to minimize bias and strengthen the validity of the findings. Our literature search will be conducted across multiple databases to ensure the inclusion of relevant studies from diverse sources. We will also include studies with diverse animal models, with no restriction on the specific species or strains of the animals used nor on the specific visceral disease induced, to ensure a comprehensive overview of the topic. Independent screening and data extraction by two investigators with arbitration procedures for unresolved discrepancies will ensure consistency and reliability.

There are, however, limitations in the proposed review. Our literature search will be limited to the English language due to resource and translation constraints, which may introduce language bias and limit the comprehensiveness of the review. Grey literature (eg, conference abstracts, theses, preprints) will be also excluded, as these sources often lack sufficient detail for accurate data extraction and quality assessment; however, this exclusion may introduce publication bias. The review will focus primarily on EB dye and equivalent markers of plasma extravasation; although this ensures methodological consistency, it may not provide a comprehensive overview of all relevant evidence relating to mechanisms of referred visceral pain. In addition, while animal studies provide essential mechanistic insights, there are fundamental differences between animal models and humans, including variations in anatomy, physiology, neurocircuitry, and disease characteristics, which may limit direct translation of findings [28,29].

In summary, referred visceral pain remains a complex and incompletely understood phenomenon. While several preclinical studies have identified cutaneous neurogenic inflammation as a potential peripheral mechanism contributing to somatic manifestations of visceral disease, no systematic review has been conducted to summarize and evaluate the rigor of relevant studies. The proposed review will address this gap by providing the first comprehensive synthesis of preclinical findings on this topic, serving as an important step toward elucidating mechanisms underlying referred visceral pain and informing future research in this area.

## Appendix

Multimedia Appendix 1 PRISMA-P checklist.

Multimedia Appendix 2 Data extraction template.

## Abbreviations

EB: Evans Blue
PRISMA-P: Preferred Reporting Items for Systematic Review and Meta-Analysis Protocols
RoB: Risk of Bias
SYRCLE: Systematic Review Centre for Laboratory Animal Experimentation

## References

[1] Lai HH, Gardner V, Ness TJ, Gereau RW (2014). Segmental hyperalgesia to mechanical stimulus in interstitial cystitis/bladder pain syndrome: evidence of central sensitization. J Urol.

[2] Farrell KE, Callister RJ, Keely S (2014). Understanding and targeting centrally mediated visceral pain in inflammatory bowel disease. Front Pharmacol.

[3] Gornet ME, Chandawarkar A, Herati A, Dellon AL (2021). Interstitial cystitis/bladder pain syndrome as referred pain from injured T12/L1 nerves: symptomatic improvement with resection of ilioinguinal and iliohypogastric nerves. Urology.

[4] Foreman R, Garrett K, Blair R (2015). Mechanisms of cardiac pain. Compr Physiol.

[5] van Oosterhout REM, de Boer AR, Maas AHEM, Rutten FH, Bots ML, Peters SAE (2020). Sex differences in symptom presentation in acute coronary syndromes: a systematic review and meta‐analysis. JAHA.

[6] Sikandar S, Dickenson A (2012). Visceral pain: the ins and outs, the ups and downs. Curr Opin Support Palliat Care.

[7] Luz L, Fernandes E, Sivado M, Kokai E, Szucs P, Safronov B (2015). Monosynaptic convergence of somatic and visceral C-fiber afferents on projection and local circuit neurons in lamina I: a substrate for referred pain. Pain.

[8] Beissner F, Henke C, Unschuld PU (2011). Forgotten features of head zones and their relation to diagnostically relevant acupuncture points. Evid Based Complement Alternat Med.

[9] Arendt-Nielsen Lars, Schipper K, Dimcevski G, Sumikura H, Krarup AL, Giamberardino MA, Drewes AM (2008). Viscero-somatic reflexes in referred pain areas evoked by capsaicin stimulation of the human gut. Eur J Pain.

[10] Van Oudenhove L, Kragel PA, Dupont P, Ly HG, Pazmany E, Enzlin P, Rubio A, Delon-Martin C, Bonaz B, Aziz Q, Tack J, Fukudo S, Kano M, Wager TD (2020). Common and distinct neural representations of aversive somatic and visceral stimulation in healthy individuals. Nat Commun.

[11] Johns E, Tracey I (2009). Neuroimaging of visceral pain. Rev Pain.

[12] Chang L (2005). Brain responses to visceral and somatic stimuli in irritable bowel syndrome: a central nervous system disorder?. Gastroenterol Clin North Am.

[13] Jin Q, Chang Y, Lu C, Chen L, Wang Y (2023). Referred pain: characteristics, possible mechanisms, and clinical management. Front Neurol.

[14] Wesselmann U, Lai J (1997). Mechanisms of referred visceral pain: uterine inflammation in the adult virgin rat results in neurogenic plasma extravasation in the skin. Pain.

[15] Fang Y, Han S, Li X, Xie Y, Zhu B, Gao X, Ma C (2021). Cutaneous hypersensitivity as an indicator of visceral inflammation via c-nociceptor axon bifurcation. Neurosci Bull.

[16] Fan Y, Ryu Y, Zhao R, Bills KB, Steffensen SC, Yang CH, Kim HY (2020). Enhanced spinal neuronal responses as a mechanism for increased number and size of active acupoints in visceral hyperalgesia. Sci Rep.

[17] He W, Wang X, Shi H, Bai W, Cheng B, Su Y, Yu X, Jing X, Zhu B (2017). Cutaneous neurogenic inflammation in the sensitized acupoints induced by gastric mucosal injury in rats. BMC Complement Altern Med.

[18] Rong P, Li S, Ben H, Li L, Yu L, Cui C, Li X, Zhu B (2013). Peripheral and spinal mechanisms of acupoint sensitization phenomenon. Evid Based Complement Alternat Med.

[19] Kim D, Ryu Y, Hahm DH, Sohn BY, Shim I, Kwon OS, Chang S, Gwak YS, Kim MS, Kim JH, Lee BH, Jang EY, Zhao R, Chung JM, Yang CH, Kim HY (2017). Acupuncture points can be identified as cutaneous neurogenic inflammatory spots. Sci Rep.

[20] Kim H, Hahm D, Sohn B, Choi Y, Pyun K, Lee H, Shim I (2006). Skin on GV01 acupoint in colonic inflammatory states: tenderness and neurogenic inflammation. J Physiol Sci.

[21] Gee MD, Lynn B, Cotsell B (2004). The relationship between cutaneous C fibre type and antidromic vasodilatation in the rabbit and the rat. J Physiol.

[22] Sorkin LS, Eddinger KA, Woller SA, Yaksh TL (2018). Origins of antidromic activity in sensory afferent fibers and neurogenic inflammation. Semin Immunopathol.

[23] Shamseer L, Moher D, Clarke M, Ghersi D, Liberati A, Petticrew M, Shekelle P, Stewart LA, PRISMA-P Group (2015). Preferred reporting items for systematic review and meta-analysis protocols (PRISMA-P) 2015: elaboration and explanation. BMJ.

[24] de Vries RBM, Hooijmans CR, Langendam MW, van Luijk J, Leenaars M, Ritskes‐Hoitinga M, Wever KE (2015). A protocol format for the preparation, registration and publication of systematic reviews of animal intervention studies. Evid Based Preclin Med.

[25] Wick MJ, Harral JW, Loomis ZL, Dempsey EC (2018). An optimized Evans blue protocol to assess vascular leak in the mouse. JoVE.

[26] Radu M, Chernoff J (2013). An in vivo assay to test blood vessel permeability. J Vis Exp.

[27] Hooijmans CR, Rovers MM, de Vries RB, Leenaars M, Ritskes-Hoitinga M, Langendam MW (2014). SYRCLE's risk of bias tool for animal studies. BMC Med Res Methodol.

[28] Van Norman GA (2019). Limitations of animal studies for predicting toxicity in clinical trials: is it time to rethink our current approach?. JACC Basic Transl Sci.

[29] Domínguez-Oliva Adriana, Hernández-Ávalos Ismael, Martínez-Burnes Julio, Olmos-Hernández Adriana, Verduzco-Mendoza A, Mota-Rojas D (2023). The importance of animal models in biomedical research: current insights and applications. Animals (Basel).

