# Supplementary material for: Inside-Out Mechanism of Referred Visceral Pain: Protocol for a Systematic Review of Cutaneous Neurogenic Inflammation in Preclinical Animal Models of Visceral Diseases
Source: JMIR Res Protoc. 2026 Apr 17;15:e67852. doi: 10.2196/67852 (PMC13135156; doi:10.2196/67852)
Supplement: Multimedia Appendix 2 [file resprot_v15i1e67852_app2.pdf]

Multimedia Appendix 2. Data Extraction Template

**Category**	**Data Item**	**Description/Details to Extract**
1. Publication detail	Author	Name of the first author(s)
	Title	Full title of the publication
	Year	Year study was published
	Journal	Journal name
	Country	Country where the study was conducted
2. Study population	Species/strain	Animal species and specific strain used
	Sex	Specify male, female, or both
	Age/Weight	Mean or range, as reported
3. Visceral disease model	Target organ	Organ or system affected (e.g., colon)
	Disease model	Name of the disease (e.g., colitis)
	Method of disease induction	Type of disease induction method (e.g., chemical, surgical); Name of inducing agent and dosage, if applicable; Duration/time course, if available
	Sample size	Total number of animals in the disease group
4. Comparison or control group	Control type	Type of control or comparison (e.g., sham, vehicle, untreated)
	Method of control	Description of how control condition was established (e.g., saline injection)
	Sample size	Total number of animals in the control group
5. Outcome – cutaneous neurogenic inflammation (primary)	EB dye or equivalent	Description of methods as reported (route, dosage, and timing of EB injection)
	Quantification method	Method of assessing EB extravasation as reported (e.g., visual count)
	Distribution	Location and pattern of EB extravasation
	Extent/Intensity	Number or density of EB extravasation in disease vs. control (include p-value, if reported)
6. Outcome – pain sensitivity (secondary)	Pain Assessment	Y/N; if reported, describe methods used (e.g., von Frey test) and locations assessed
	Key finding	If applicable, summary of pain-related outcomes
7. Other relevant findings	Other lab techniques	If applicable, describe other techniques/approaches used to characterize the specific location on the body surface showing EB extravasation (e.g., immunohistochemistry)
	Findings	Corresponding findings for each technique
8. Acupuncture point-related information (if applicable)	Findings	Describe relevant findings (e.g., name of acupuncture point locations showing EB extravasation)
